# Role of ErbB Receptors in Cancer Cell Migration and Invasion

**DOI:** 10.3389/fphar.2015.00283

**Published:** 2015-11-24

**Authors:** Aline Appert-Collin, Pierre Hubert, Gérard Crémel, Amar Bennasroune

**Affiliations:** ^1^UMR CNRS 7369, Unité Matrice Extracellulaire et Dynamique Cellulaire, Université de Reims Champagne-ArdenneReims, France; ^2^Laboratoire d’Ingénierie des Systèmes Macromoléculaires, CNRS-AMU UMR 7255Marseille, France; ^3^INSERM U1109 MN3TStrasbourg, France; ^4^UMR CNRS 7360, Laboratoire Interdisciplinaire des Environnements Continentaux, Université de LorraineMetz, France

**Keywords:** ErbB receptors, cancer, epithelial-mesenchymal transition, migration, cell signaling

## Abstract

Growth factors mediate their diverse biologic responses (regulation of cellular proliferation, differentiation, migration and survival) by binding to and activating cell-surface receptors with intrinsic protein kinase activity named receptor tyrosine kinases (RTKs). About 60 RTKs have been identified and can be classified into more than 16 different receptor families. Their activity is normally tightly controlled and regulated. Overexpression of RTK proteins or functional alterations caused by mutations in the corresponding genes or abnormal stimulation by autocrine growth factor loops contribute to constitutive RTK signaling, resulting in alterations in the physiological activities of cells. The ErbB receptor family of RTKs comprises four distinct receptors: the EGFR (also known as ErbB1/HER1), ErbB2 (neu, HER2), ErbB3 (HER3) and ErbB4 (HER4). ErbB family members are often overexpressed, amplified, or mutated in many forms of cancer, making them important therapeutic targets. EGFR has been found to be amplified in gliomas and non-small-cell lung carcinoma while ErbB2 amplifications are seen in breast, ovarian, bladder, non-small-cell lung carcinoma, as well as several other tumor types. Several data have shown that ErbB receptor family and its downstream pathway regulate epithelial-mesenchymal transition, migration, and tumor invasion by modulating extracellular matrix (ECM) components. Recent findings indicate that ECM components such as matrikines bind specifically to EGF receptor and promote cell invasion. In this review, we will present an in-depth overview of the structure, mechanisms, cell signaling, and functions of ErbB family receptors in cell adhesion and migration. Furthermore, we will describe in a last part the new strategies developed in anti-cancer therapy to inhibit ErbB family receptor activation.

## Structure of ErbB Family Receptors

**FIGURE 1:**
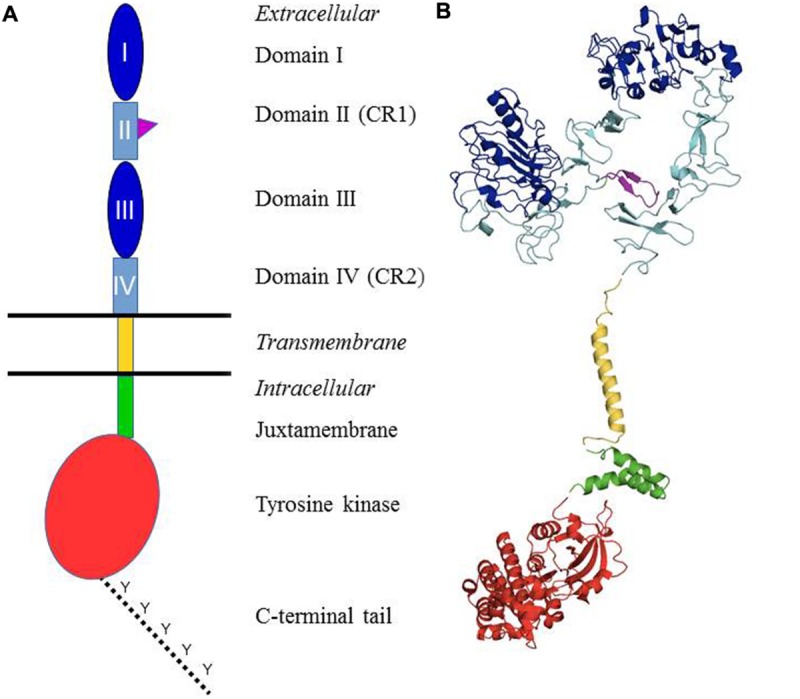
**Structural organization of the ErbB/HER receptors. (A)** Shows a schematic representation of the different domains. The extracellular part of the receptors is composed of four domains: I and III = ligand-binding domain (dark blue); II and IV = cysteine-rich domains (light blue). The domain II contains the dimerization arm (purple). It is followed by the single transmembrane domain (yellow), a juxtamembrane domain (green), the tyrosine kinase domain (red) and a C-terminal tail which contains the main tyrosines that are phosphorylated upon receptor activation (dotted line). **(B)** Presents the structures of different domains of the human EGFR which have been established through X-ray crystallography or solution RMN. Color coding is identical to **(A)**. Protein Data Bank (PDB) accession codes are as follows: ectodomain (in closed unliganded conformation), 3QWQ, transmembrane region, 2KS1, juxtamembrane domain, 1Z9I; kinase domain, 3W32. No structure is available for the C-terminal tail.

Epidermal growth factor (EGF) was one of the first growth factors discovered in the early 1960s. It was shown to be a polypeptide able to stimulate growth and differentiation of cells of epidermal and mesodermal origin (Cohen, 1983). Subsequent studies identified the receptor and the receptor’s intrinsic kinase activity. EGF was shown to bind with high affinity to a specific receptor located in the cell membrane and stimulate rapid activation of a protein kinase activity. The EGFR was purified and characterized as a ∼170 kDa molecular weight integral membrane glycoprotein, bearing ligand-inducible kinase activity. The EGFR kinase activity was shown to result in phosphorylation of tyrosine residues, the first such demonstration for any receptor. It was also found that ligand binding induces receptor clustering and that antibody cross-linking mimics the effects of EGF, indicating the importance of receptor dimerization/oligomerization in its activation. Cloning of the human receptor was performed in Ullrich et al. (1984). Analysis of the sequence confirmed previous data, confirming the glycoprotein nature of EGFR, and the presence of a tyrosine-specific protein kinase sequence. Molecular cloning of EGFR also revealed a close similarity with the viral v-erbB oncogene, yielding the first indication of a link between growth factor receptors and cancer. Cloning techniques also revealed the existence of three related membranes receptors, which were called ErbB2–4, or Human EGF Receptor (HER) and share the overall primary structure of EGFR. Furthermore, during activation mechanism, the four members of the family can form various heterodimers, potentially yielding a wide array of signaling outcomes. Subsequent work allowed for a more precise delineation of the different domains composing the receptor (**Figure 1A**) (Ceresa and Peterson, 2014; Roskoski, 2014).

Based upon the primary amino acid structure of EGFR, the four ErbB receptors consist of a large extracellular domain, a single hydrophobic transmembrane segment, and an intracellular domain consisting of a juxtamembrane domain, a typical tyrosine protein kinase segment, and a tyrosine-rich carboxyterminal tail. Upon receptor activation, a number of these C-terminal tyrosines are phosphorylated. The extracellular domain itself is made of a tandem repeat of two types of subdomains: domains I and III, which are leucine-rich segments that make up the ligand binding, and cysteine-rich domains II and IV. Domain II participates in homo and heterodimer formation with ErbB family members (see below).

**FIGURE 2:**
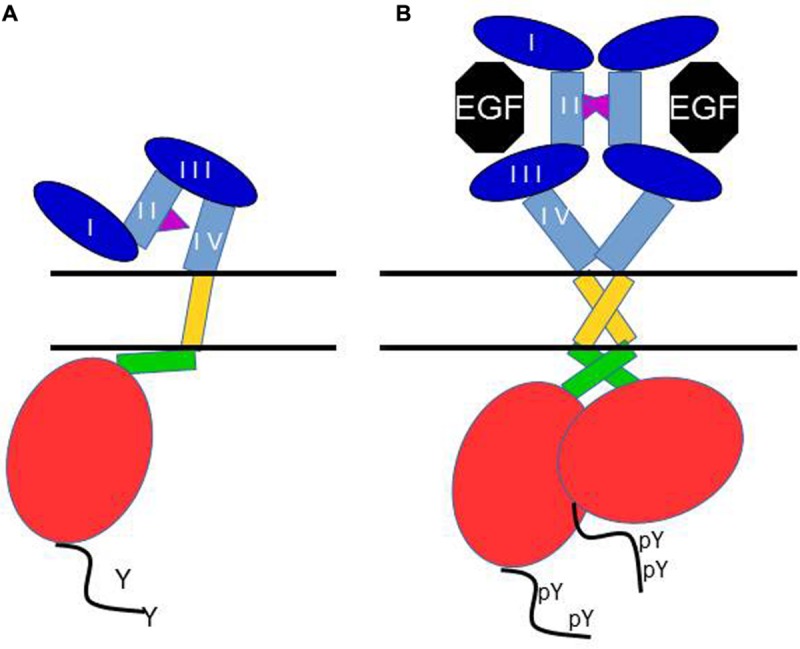
**Schematic of the current view of main structural events in the activation of the EGF receptor.** In **(A)** the receptor is depicted in its monomeric, unliganded, inactive form. The dimerization arm of the extracellular domain II binds to domain IV, and the juxtamembrane domain interacts with membrane phospholipids. In **(B)** binding of EGF to one monomer to domains I and III induces a conformation change which makes the dimerization arm available for interaction with another extended ligand-bound monomer, and causes dimerization. This conformational change is accompanied by the formation of an anti-parallel interaction between the two juxtamembrane domains, and thus an asymmetric “head to tail” interaction of the two kinase domains, resulting in allosteric activation of the kinase, and C-terminal tail tyrosine phosphorylation.

The first crystallographic view of the EGFR kinase domain confirmed its likeness with previous published protein kinase structures, with two lobes defining an ATP-binding cleft. But this provided little insight into how the kinase is activated by receptor dimerization. Analysis of additional crystal structures of the active EGFR kinase domain revealed a characteristic asymmetric dimer (Zhang et al., 2006). In this dimer, the large carboxy lobe of one kinase binds the small amino lobe of the other kinase domain (**Figure 2B**). This is reminiscent of the activation mechanism of the cyclin-dependent kinases (CDKs) and Src family kinases.

NMR structures have also been published for the dimeric transmembrane segments of the ErbB receptors, and their importance in the ligand-induced activation of the EGFR has been confirmed very recently in a series of papers by the Kuriyan group (reviewed in Endres et al., 2014). In this work, an assymetric interaction of the juxtamembrane domains was also described upon ligand-induced rearrangements of the receptor structure, leading to kinase domains interactions and activation.

### Regulation of ErbB Activity: Mechanisms of Dimerization and Activation

As previously described (see above), in the general case, growth factors bind RTKs, as ErbB receptors, which induce their dimerization and subsequent activation (Ullrich and Schlessinger, 1990). A multitude of extracellular polypeptide ligands can bind ErbB receptors. Indeed, numerous growth factors have been described as ligands for this receptor family, and these polypeptides are divided into four group. EGF receptors bind to EGF, epigen, transforming growth factor, and amphiregulin. Betacellulin, heparin-binding EGF-like growth factor, and epiregulin bind to EGFR and ErbB4. The third group -which binds to ErbB3 and ErbB4- includes neuregulin-1 and neuregulin-2. The last group of ligands binds to ErbB4 and consists of neuregulin-3 and neuregulin-4 (Roskoski, 2014). ErbB2 has no known ligand. These ligands exist usually in a proform as transmembrane precursors which submit a proteolytic processing to release the soluble, active N-terminal ectodomains (Massague and Pandiella, 1993; Singh and Harris, 2005).

Deeper insights in the structure-function relationship of the ErbB receptors were published in the early 2000s with a series of crystal structures of the EGFR extracellular region with and without bound ligand (**Figure 1B**). The EGFR extracellular region dimerization is mediated entirely by receptor–receptor contacts, by a “dimerization arm” that projects from domain II (Lemmon and Schlessinger, 2010). In fact, two different conformational states have been described for the extracellular region of EGFR, ErbB3, and ErbB4 (**Figure 2**) (Cho and Leahy, 2002; Bouyain et al., 2005; Dawson et al., 2005). The first one is the inactive conformation: in the absence of ligand, the EGFR adopts a monomeric, compact, “tethered” conformation (**Figure 2A**) which presents an intramolecular tether between domains II and IV of the extracellular region (**Figure 2A**). This autoinhibited state prevents interaction between subregions I and III to form a ligand-binding pocket which holds the extracellular domain in a closed conformation (Riese et al., 2007; Fuller et al., 2008). In the active conformation, the crystal structures reveal a dimeric, extended conformation where the ligand bridges domains I and III, thereby opening the structure. The subregions I and III rearrangement results in the ligand-binding pocket formation that permits interactions between a single ligand molecule and these domains I and III (**Figure 2B**) (Burgess et al., 2003; Jorissen et al., 2003). The main focal point of movement resides in a “hinge” domain at the junction of domains II and III (Burgess et al., 2003). Thus, in the presence of ligand, there are no more intramolecular interaction between domains II and IV, resulting in exposure of the dimerization arm of domain II. This allows receptor dimerization *via* intermolecular contacts that involve mostly the dimerization arm in subregion II (**Figure 2B**). A small region, C-terminal of the dimerization arm, in domain II as well as part of domain IV are also involved in the dimerization, albeit to a lesser extent (Dawson et al., 2005). ErbB2 differs significantly from this scheme, in that it has no known ligands, but the structure of its extracellular domain shows an extended configuration, seemingly poised for hetero-interactions with other ErbB family members.

Thus, the model for receptor activation which has been proposed is as follows: unliganded EGFR, ErbB3 and ErbB4 receptors exist in an autoinhibited form that undergoes domain rearrangement to an active form after ligand binding. This rearrangement juxtaposes domains I and III breaking the domain II–IV tether and unmasking the domain II to participate in receptor dimerization and activation of signal transduction.

After homo- or heterodimerization, the activation of intrinsic protein kinase activity at the intracellular c-terminus results in the stimulation of the intrinsic catalytic activity of the receptor and phosphorylation of specific tyrosine residues of the receptors (Bennasroune et al., 2004b). These molecular mechanisms associated with RTK activation have been described by biochemical and structural studies, and imply structural modifications (Hubbard, 1999; Hubbard and Till, 2000). The precise molecular mechanism vary somewhat between the different families of RTKs. In many cases (insulin receptor, Eph, PDGF receptor, …), it is the autophosphorylation of an activation loop in the kinase domain which is responsible for the transition to the active kinase conformation. This is not the case for ErbB receptors for which the transition to the active form is rather due to the formation of an asymmetric dimer of the kinase domains, in which one kinase allosterically activates the other one. The kinase domains then catalyze the phosphorylation of tyrosine residues (outside the kinase domain in the C-terminal tail) creating docking sites for adaptor proteins or enzymes involved in downstream signal transduction.

Several downstream signaling pathways are activated after specific ErbB receptor activation (by homo- or heterodimerization) resulting notably in actin polymerization and intracellular organization necessary for migration and invasion of epithelial cells (Feigin and Muthuswamy, 2009). When ligands bind to ErbB receptors, they trigger a cascade of biochemical events inducing stimulation of rich signaling pathways. This intracellular signaling involves a variety of molecules known as adaptors and scaffolding proteins (Pawson and Scott, 1997). For example, Grb2 is an important adaptor in the activation of the ras/raf/MAPK pathway. These adaptors often feature several motifs that mediate interactions between intracellular proteins: Phosphotyrosine-binding (PTB) and Src homology 2 (SH2) domains specifically bind to phosphotyrosine, whereas SH3 domain binds to proline-rich sequences of target proteins. Thus, these adaptor molecules permit to recruit specific proteins to establish signaling networks particular to a cascade and a cell location.

Among these signaling cascades, ErbB receptor activation is associated (i) with the phosphatidylinositol 3-kinase (PI3K)/Akt (PKB) pathway which plays a key role in cell survival, (ii) and with the Ras/Raf/MEK/ERK1/2 and the phospholipase C (PLCγ) pathways mediating cell proliferation (Yarden and Pines, 2012). In the following chapter, we will focus on the role of ErbB family receptors in epithelial-mesenchymal transition (EMT), migration, and tumor invasion of cancer cells.

## Role of ErbB Receptors in Cancer and New Strategies Developed in Anti-Cancer Therapy

ErbB receptors were linked to human cancer pathogenesis by about three decades ago. For example, EGFR and ErbB2 are mutated in many epithelial tumors and clinical studies suggest that they play an important role in cancer development and progression. These receptors have been largely studied, not only to understand the mechanisms underlying their oncogenic potential, but also to exploit them as putative therapeutic targets. In this part, we will focus on the role of ErbB receptors in EMT, migration and tumor invasion. Then, we will summarize the new therapeutic approaches to inhibit ErbB receptor activation in cancer.

### Role in Epithelial-mesenchymal Transition, Migration, and Tumor Invasion by Modulating Extracellular Matrix Components

ErbB receptors influence cell proliferation, differentiation, and migration. Not surprisingly, alterations of ErbB familly play a role in the development and progression of several epithelial tumors (Yarden and Sliwkowski, 2001). Cancer cell migration and invasion allow tumor spread into surrounding tissues and circulation which generates metastasis, a significant hallmark of poor prognosis (Friedl and Wolf, 2003). Overexpression of EGF and its receptors has been demonstrated in many breast cancers and was associated with a higher incidence of distant metastases (De Luca et al., 2008; Giltnane et al., 2009).

Two types of cell migration exist: mesenchymal- and amoeboid-type migration (Friedl and Wolf, 2003). Mesenchymal-type cell migration is characterized by protrusion formation such as filopodia and lamellipodia at the leading edge of migrating cells and by adhesions of these protrusions linking the actin cytoskeleton to the extracellular matrix (ECM; Parsons et al., 2010). Adhesion disassembly at the cell rear and the contraction of actomyosin then allows the cell to achieve cellular movement (Parsons et al., 2010). In carcinoma, the most prevalent form of all human cancers (80–90%), malignant transformation is associated with the loss of differentiated epithelial characteristics and a coinciding increase of less-mature mesenchymal characters during EMT. In cancer, EMT induces tumor progression by affording properties such as invasiveness, the ability to metastasize, resistance to therapy, and possibly the generation of stem-like cancer cells (Mallini et al., 2014).

Members of the ErbB receptor family play prominent roles during carcinogenesis, and most induce EMT when overexpressed both *in vitro* and *in vivo* (Al Moustafa et al., 2012). In line with the hypothesis that EGF family members play a fundamental role in the initial steps of EMT, transformation by HER2/neu resulted in increased CD44high/CD24low immortalized human mammary epithelial cells with many of the stem-like properties of the initial steps of EMT (Morel et al., 2008). In oral squamous cell carcinoma cells, inhibition of EGFR induced a transition from a fibroblastic morphology to a more epithelial phenotype with an accumulation of desmosomal cadherins at cell–cell junctions (Lorch et al., 2004). These studies suggest that EGFR signaling mediates the initial steps of EMT, and that EGFR inhibition may restrain EMT in some cellular contexts. In fact, ligand-independent, constitutively active forms of EGFR can increase motility and invasiveness of tumor cells, and EGFR inhibitors block cancer cell migration *in vitro*. Cellular migration and invasion is inhibited by blocking EGFR and consequently its pathways, by a monoclonal antibody (mAb) or a tyrosine kinase inhibitor (TKI), suggesting a crucial role for EGFR inhibitors in the control of cancer metastasis (Yue et al., 2012; Liu et al., 2014). Furthermore, Jeon et al. (2015) demonstrated that HER2 expression level plays an important role in the induction of fibronectin expression, a major component of ECM, in breast cancer cells that triggers cell adhesion and cell invasion.

Several studies have revealed that HER2 is expressed in circulating tumors cells of early and metastatic breast cancer patients. The consequences of HER2 expression are usually more severe in circulating tumors cells in comparison to the corresponding primary tumors (Kallergi et al., 2008; Fehm et al., 2010). Indeed, circulating tumor cells and metastases of breast cancer present a dynamic *in vivo* pattern of EMT (Bonnomet et al., 2012). CD44^+^/CD24^-^ subpopulation of tumor cells which overexpressed RAS or HER2 have a phenotype with increased EMT potential (Wang et al., 2012; Bhat-Nakshatri et al., 2013). The CD44^+^/CD24^-/low^ gene expression signature, identified as a “claudin-low” molecular subtype (Creighton et al., 2009), is characterized by expression of many EMT-associated genes, such as FoxC2, Zeb, and N-cadherin (Morel et al., 2008; Creighton et al., 2009). EMT in breast cancer stem cells could play an important role in the metastatic phenomenon (Korkaya et al., 2012; Wang et al., 2012). Furthermore, several studies have highlighted that HER2 regulates the stem cell population and then contributes to mammary carcinogenesis (Bedard et al., 2009) and that HER2 overexpression in multiple breast cancer cell lines results in an increase of ALDH1^+^ cell fraction, which has a greater capacity to invade and form tumors in immunodeficient mice (Korkaya et al., 2008).

Integrins, focal adhesion kinase (FAK), and Rho GTPases (Rho, Rac, Cdc42) are important regulators in mesenchymal-type migration (Parsons et al., 2010) and may be influenced by EGFR signaling. Indeed, ErbB signaling induces cell adhesion and migration by modulation of e.g., FAK or Rho GTPases (Fichter et al., 2014). For example, Fichter et al. (2014) showed that inhibition of EGFR signaling in esophageal squamous cell carcinomas rearranges the actin cytoskeleton, induces focal adhesions, and limits esophageal cancer cell migration by rapid inhibition of ERK1/2, Akt, STAT3, and RhoA activity. However, as (i) Zhan et al. have shown that only EGFR/ErbB2 heterodimers increased the invasive potential of mammary epithelial cells which is not observed with homodimers (Zhan et al., 2006), and as (ii) Guy et al. (1992) described that EGFR overexpression in mice was not associated with transformation of the entire mammary epithelium, but provoked only focal mammary tumors (sometimes metastatic), these results suggest that additional mechanisms are probably involved in ErbB activation effects on EMT and cell invasion.

Cancer cells secrete EGF-like, growth factors that can play a role directly on endothelial cells (Kuo et al., 2012). The microenvironment can also act on tumor cells. Indeed, EGF-like peptides and angiogenic growth factors that can both act on endothelial cells and activate EGFR in cancer cells are produced by bone marrow stromal cells (Fidler, 2002). EGFR activation in human carcinoma cell lines also increases matrix metalloproteinase-9 (MMP-9) activity, which increases *in vitro* cell invasion by facilitating disintegration of ECM barriers to tumor invasion (Zuo et al., 2011).

During the mesenchymal mode of invasion, the presence of proteases in ECM that can degrade the surrounding ECM (Sahai and Marshall, 2003; Wolf et al., 2003) will cause the liberation of small peptides originating from the fragmentation of ECM proteins. These molecules called matrikines limit EGFR signaling to the perimembrane area of the cytosol, a mode that is preferential for motility (Iyer et al., 2008) and cell survival (Fan et al., 2007; Rodrigues et al., 2013). Tenascin C (TNC) establishes interactions between the epithelium and the mesenchyme during embryonic development, tissue differentiation and wound repair but persistent high levels of TNC are present in various tumor tissues, including brain, bone, prostate, intestine, lung, skin, and breast (Pas et al., 2006). TNC is a hexameric glycoprotein of which each subunit contains: the N-terminal assembly domain, a domain composed of 14.5 EGF-like repeats (EGFL), a domain composed of a varied number of fibronectin type III-like repeats, and a fibrinogen-like sequence on the C terminus (Orend and Chiquet-Ehrismann, 2006). The EGF-like repeats of TNC also have counter-adhesive properties (Spring et al., 1989; Prieto et al., 1992) and have been shown to bind and signal through the EGFR (Swindle et al., 2001; Iyer et al., 2007). Interestingly, the binding of TNC EGFL to EGFR preferentially promotes cell migration by limiting receptor signaling to the perimembrane space (Iyer et al., 2008). Indeed, the binding of TNC EGFL to the receptor does not induce ligand-induced internalization of the receptor (Iyer et al., 2007). Thus, essentially all of the EGFR signaling occurs from the plasma membrane locale. Based on the results obtained on the signaling, authors propose that plasma membrane-associated signaling of EGFR is preferential for motility. Others matrikines derived from Thrombospondin 1 and Laminin-332 feature EGFL domains that have been shown to bind and activate EGFR (Schenk et al., 2003; Liu et al., 2009).

This section has highlighted the importance of the ErbB family receptors in regulating EMT during cancer. Understanding and defining the initial molecular signals leading to the EMT switch in tumor cells would absolutely participate to the earliest possible clinical detection and therapeutic strategies. While the use of inhibitors delivered individually to ErbB/EGF targets seems reasonable, limited effect suggests that a combinatorial approach could permit substantial improvements in clinical outcome. Enlightening the steps that induce the re-activation of embryonic processes and signaling pathways in cancer, such as those involved in EMT, and best understanding the interactions between cells and their microenvironment, will lastly lead to more rational strategies in our arsenal for targeting cancer.

### New Strategies Developed in Anti-cancer Therapy to Inhibit ErbB Family Receptor Activation

Advances in genetic engineering and fundamental research applied to a better understanding of the biology of ErbB signaling in cancer have led to the development of many therapeutic agents including monoclonal antibodies (mAbs), small-molecule TKIs and other agents like peptides, affibodies, nanobodies, etc. (Bennasroune et al., 2004b; Alaoui-Jamali et al., 2015). In this paragraph, we present a partial overview of current development of drugs targeting ErbB receptors. **Table 1** presents several examples of drugs, their targets and their current status in term of clinical trials.

**Table 1:** Some examples of drugs targeting ErbB receptor family.

Drug	Company	Receptor	Description	Status	Indication
Cetuximab (Erbitux)	ImClone Systems	EGFR	mAb directed against EGFR	First approval by FDA in 2004	Colorectal, head, neck and pancreas cancers
Panitumumab (Vectibix)	Amgen	EGFR	mAb directed against EGFR	First approval by FDA in 2006	Metastatic colorectal cancer
Erlotinib (Tarceva)	Roche/Genentech/OSI	EGFR	Inhibitor of EGFR signaling	First approval by FDA in 2004	Non-small cell lung cancer, pancreatic cancer
Gefitinib (Iressa)	AstraZeneca	EGFR	Inhibitor of EGFR signaling	First approval by FDA in 2003	Non-small cell lung cancer, esophageal cancer
Lapatinib (Tykerb/Tyverb)	GlaxoSmithKline	EGFR/HER2	Inhibitor of EGFR/HER2 signaling	First approval by FDA in 2007	Metastatic breast cancer
Dacomitinib	Pfizer	EGFR/HER2/HER4	Pan-inhibitor of ErbB receptors signaling	Phase III	Non-small cell lung cancer
Trastuzumab (Herceptin)	Genentech	HER2	mAb directed against HER2	First approval by FDA in 1998	HER2-positive breast cancer HER2-overexpressing metastatic gastric or gastroesophageal junction adenocarcinoma
Pertuzumab (Perjeta)	Genentech	HER2	mAb directed against HER2	First approval by FDA in 2012	Breast cancer
Margetuximab (MGAH22)	MacroGenics	HER2	mAb directed against HER2	Phase I	Breast, gastroesophageal and other HER2-positive tumors
Patritumab (U3-1287)	Daiichi Sankyo Pharmaceutical Development and Amgen	HER3	mAb directed against HER3	Phase I–II	Non-small cell lung cancer

*mAb, monoclonal antibody.*

Cetuximab and Panitumumab are mAbs that bind to EGFR, possessing anti-tumor activity. They are frequently used in treatment of metastatic colorectal and head/neck cancer. Cetuximab is a chimeric human: murine immunoglobulin G1 (IgG1) mAb. It binds to EGFR with higher affinity that EGF (Kim et al., 2001) and also binds to the mutant receptor EGFRvIII. The cetuximab promotes EGFR internalization (Sunada et al., 1986). Panitumumab (ABX-EGF), fully human mAb with high EGFR affinity, blocks ligand-binding and induces EGFR internalization (Yang et al., 2001). Activity of Panitumumab has been demonstrated against variety of advanced cancer patients, including renal carcinomas and metastatic colorectal cancer in clinical trials (Douillard et al., 2010). Another antibody against a second member of ErbB receptor family has been developed. Trastuzumab or Herceptin selectively binds to the extracellular domain of HER2 receptors and inhibits downstream signaling pathways. This inhibition results in decreased proliferation of tumor cells. Trastuzumab identifies tumor cells for immune destruction, and then, promotes an antibody-dependent cellular cytotoxicity, causing apoptosis of tumor cells (Molina et al., 2001). Trastuzumab is predominantly used in the treatment of the ErbB2-positive breast cancer subtype where its combination with conventional chemotherapy, had a significant effect on disease free survival of patients with early stage ErbB2+ breast cancer (Hudis, 2007).

Currently, small molecule inhibitors under clinical trials or approved by the US Food and Drug Administration (FDA) are reversible or irreversible inhibitors. They bind to the ATP-binding site in the kinase domain of ErbB receptors and next inhibit their intracellular kinase activity. Most of the existing small molecule TKIs which target RTK are multi-targeted and inhibit a variety of molecules in a non-specific manner. This characteristic has been demonstrated to have several disadvantages. It’s why only a few specific and selective TKIs have been approved by authorities for cancer treatment. Several approaches have been developed: TKI that targets a specific member of the ErbB family or TKI that inhibit multiple members of the ErbB family. These last inhibitors bind their targets irreversibly and are currently under evaluation for the treatment of cancer. Erlotinib or gefitinib are the first-generation reversible EGFR-TKIs and are approved first-line therapies for patients with non-small cell lung cancer (NSCLC) presenting activating EGFR mutations. Unfortunately, despite these agents have demonstrated improvement in progression-free survival, patients present resistance to these agents and tumors rapidly regrow (Hirsh, 2015).

Lapatinib (Tykerb/Tyverb^®^), developed in Xia et al. (2002), is an orally active reversible dual TKI of EGFR/HER2. It binds covalently to the Cys 773 of EGFR and Cys 805 of HER2 (Howe and Brown, 2011). Studies *in vitro* and *in vivo* using xenografted mice with cell lines over-expressing EGFR and HER2 have shown that lapatinib inhibits tyrosine phosphorylation in catalytic domain of EGFR and HER2 and prevents ERK1/2 and AKT activation which induces apoptosis of tumor cells (Xia et al., 2002). In 2007, Lapatinib was approved by the FDA for patients with breast cancer as second-line treatment. Lapatinib, in combination with an aromatase inhibitor, was also used as first-line therapy for treatment of postmenopausal women with estrogen/HER2 receptor-positive breast cancer. Another inhibitor, Dacomitinib (PF-00299804, PF299), is currently under development. Dacomitinib is a selective, quinazalone-based irreversible pan-HER inhibitor of EGFR/ErbB1, ErbB2/HER2, and ErbB4/HER4–TKI and is in phase III of clinical development for the treatment of NSCLC. Furthermore, other small molecule inhibitors targeted against ErbB receptors are currently in clinical trials (Hirsh, 2015).

Recently, anticancer strategies that involve smaller antibody fragments such as Fragment antigen-binding domain (Fabs), single-chain variable fragment (ScFvs) and nanobodies are in development (Holliger and Hudson, 2005). Nanobodies consist of single-domain antigen-binding fragments derived from the camelids heavy-chain only antibodies (Muyldermans, 2001). Nanobodies have several advantages: they are significantly smaller in size (15 kDa) than scFv (28 kDa) or Fab (55 kDa), and then potentially providing higher tissue dispersion and superior tissue penetration than their counterparts (Holliger and Hudson, 2005); they are also significantly more stable than V_H_ (Heavy chain) domains (Stijlemans et al., 2004). Nanobodies specific for EGFR have recently been developed. They inhibit the binding of EGF to the receptor by different mechanisms either by blocking ligand binding to EGFR in a manner similar to cetuximab or by binding an epitope near the EGFR domain II/III junction and then by preventing receptor conformational changes required for high-affinity ligand binding and dimerization (Schmitz et al., 2013). Nanobodies binding to EGFR thereby inhibits EGFR signaling (Roovers et al., 2007, 2011). Several studies have shown that several EGFR-specific nanobodies have the potential to reproduce the clinical efficacy of mAbs such as cetuximab. Moreover, these molecules are more stable and less costly to produce than mAbs. In addition, potent multivalent nanobodies can be produced and can bind a number of targets (Jahnichen et al., 2010; Roovers et al., 2011), allowing to design multivalent agents that combine several modes of EGFR or other cancer target inhibition.

Affibody molecules are derived from the B-domain in the Ig-binding region of *Staphylococcus aureus* protein A (Nygren, 2008). Affibodies are highly soluble, chemically and thermally stable and rapidly removed from the circulation. The single protein chain of affibodies facilitates direct fusion with various proteins such as toxins and fluorophores, radioactive labels, or chemical groups for immobilization. The first affibody molecule, developed *in vivo*, was directed against HER2. This molecule binds to HER2 receptor on an epitope different from that of trastuzumab and with an affinity constant of 50 nM (Friedman et al., 2007). Since co-expression of HER2 and EGFR has been reported to be related with a poor prognosis in several types of cancer, a bispecific affibody directed against these receptors was generated (Friedman et al., 2009). In-depth binding studies have shown that this bispecific affibody can interact at the same time with both target receptors. Other affibody molecules which present different affinities for EGFR and HER2 were also developed to study their selectivity and their cooperativity between the two binding sites. Studies have shown that an affitoxin composed of a HER2-specific affibody linked to a truncated version of *Pseudomonas* exotoxin A was able to bind to HER2 with nanomolar affinity. This affitoxin eliminated HER2-positive cells with IC_50_ values three orders of magnitude lower than the corresponding HER2-negative cells, and induce a rapid shrinkage of BT-474 breast cancer xenograft tumors. This study demonstrates that HER2-affitoxin is an encouraging new therapeutic approach for HER2-overexpressing cancers that are non-responsive to currently available therapies (Zielinski et al., 2011).

Furthermore, new strategies has been developed this last decade to disturb ErbB receptor family dimerization and activation by targeting the transmembrane domain of these receptors: indeed, short synthetic peptides which mimick TM domains are able to inhibit specifically kinase activity and cell signaling induced by EGF and ErbB2 receptors in cancer cells (Bennasroune et al., 2004a). More recently, it has been shown that transmembrane domain targeting peptide antagonizing ErbB2/Neu exhibit anticancer properties by inhibiting breast tumor growth and metastasis in genetically engineered mouse model of breast cancer (Arpel et al., 2014).

## Conclusion

Even if the involvement of ErbB receptor family by overexpression or activating mutations in oncogenesis is well described since 25 years, numerous processes concerning the role of these proteins in dysregulation of cell proliferation and migration are not widely understood. Moreover, the cancer progression is accompanied by an extensive remodeling of ECM components. According to the current status of knowledge, several proteins of the ECM as decorin or matrikines may be used both as diagnostic markers and as targets in cancer therapy. Indeed, studying the role of ECM components and their interactions with ErbB receptors in cellular processes of growth, invasion and metastasis should permit the development of new inhibitor classes.

## Author Contributions

AB: conception and drafting of the review; AA-C: conception and drafting of the review; PH: drafting of the review and final approval; GC: Final approval of the version to be published.

## Conflict of Interest Statement

The authors declare that the research was conducted in the absence of any commercial or financial relationships that could be construed as a potential conflict of interest.
